# Functional link between mitochondria and Rnr3, the minor catalytic subunit of yeast ribonucleotide reductase

**DOI:** 10.15698/mic2019.06.680

**Published:** 2019-05-20

**Authors:** Isaac Corcoles-Saez, Jean-Luc Ferat, Michael Costanzo, Charles M. Boone, Rita S. Cha

**Affiliations:** 1 School of Medical Sciences, North West Cancer Research Institute, Bangor University, Deniol Road, Bangor, LL57 2UW, United Kingdom.; 2 Institute of Integrative Biology of the Cell (I2BC), Avenue de la Terrasse, Paris, France.; 3 University of Toronto, Donnelly Centre, 160 College Street, Toronto, Ontario, M5S 3E1, Canada.

**Keywords:** Rnr1, Rnr3, Mec1, carbon source, respiration, mitochondria, dNTP

## Abstract

Ribonucleotide reductase (RNR) is an essential holoenzyme required for *de novo* synthesis of dNTPs. The *Saccharomyces cerevisiae* genome encodes for two catalytic subunits, Rnr1 and Rnr3. While Rnr1 is required for DNA replication and DNA damage repair, the function(s) of Rnr3 is unknown. Here, we show that carbon source, an essential nutrient, impacts Rnr1 and Rnr3 abundance: Non-fermentable carbon sources or limiting concentrations of glucose down regulate Rnr1 and induce Rnr3 expression. Oppositely, abundant glucose induces Rnr1 expression and down regulates Rnr3. The carbon source dependent regulation of Rnr3 is mediated by Mec1, the budding yeast ATM/ATR checkpoint response kinase. Unexpectedly, this regulation is independent of all currently known components of the Mec1 DNA damage response network, including Rad53, Dun1, and Tel1, implicating a novel Mec1 signalling axis. *rnr3*Δ leads to growth defects under respiratory conditions and rescues temperature sensitivity conferred by the absence of Tom6, a component of the mitochondrial TOM (translocase of outer membrane) complex responsible for mitochondrial protein import. Together, these results unveil involvement of Rnr3 in mitochondrial functions and Mec1 in mediating the carbon source dependent regulation of Rnr3.

## INTRODUCTION

Ribonucleotide reductase (RNR) is a conserved holoenzyme required for *de novo* synthesis of dNTPs, the building blocks of DNA [1]. The eukaryotic RNR is a tetrameric complex composed of two large R1 catalytic subunits and two small R2 regulatory subunits. The R1 subunit contains an allosteric regulatory site that monitors the intracellular ATP/dATP ratio and controls the overall RNR catalytic activity. The R2 subunit contains a critical tyrosine residue where the radical transfer to the active site in the R1 subunit initiates [1, 2].

The genome of *Saccharomyces cerevisiae* encodes for four RNR proteins; Rnr1 and Rnr3 for the catalytic R1 subunits and Rnr2 and Rnr4 for the regulatory R2 subunits [3]. While expression of Rnr2 and Rnr4 is constitutive, Rnr1 is induced specifically during S phase. Rnr3, on the other hand, is detectable only under the condition of DNA damage or replication stress [3].

The budding yeast Mec1 is an essential serine/threonine kinase, responsible for mediating the genotoxic stress dependent induction of Rnr3 [4]. Mec1 is an ATM/ATR protein, a family of conserved phosphatidylinositol 3-kinase like kinases (PIKKs) best understood for their roles in mediating the DNA damage response (DDR) [5]. In addition, ATM/ATR proteins play critical roles in a number of fundamental processes, such as DNA replication, meiotic recombination, neuronal vesicle trafficking, and protein homeostasis [6–10]

In response to genotoxic stress, Mec1 activates RNR via two downstream kinases Rad53 and Dun1 [11]. Rad53 is an essential kinase that shares 24% and 30% identity with the human CHK1 and CHK2 kinases, respectively. The latter are ATM/ATR targets, which become activated in response to replication stress and DNA damage, respectively [5, 12]. Rad53 is phosphorylated in response to both replication stress (i.e like CHK1) and DNA damage (i.e. like CHK2) in a Mec1 dependent manner. Upon activation, Rad53 phosphorylates Dun1, which in turn activates RNR by inhibiting activities of three negative regulators; (i) Sml1, an allosteric inhibitor of Rnr1, (ii) Dif1, involved in nuclear transport of Rnr2 and Rnr4, and (iii) Rfx1/Crt1, a transcriptional repressor that downregulates *RNR3* expression [11, 13, 14].

*RNR1* and *RNR2* are essential in most yeast strain backgrounds, while inactivation of Rnr4 impacts genome duplication, DNA damage repair, and resistance to genotoxic stress. In contrast, *rnr3*Δ does not confer any obvious phenotypes, including sensitivity to replication stress or DNA damage despite the fact that its expression is massively induced during the DDR [15]. As such, the function(s) of Rnr3 remains elusive. Here, we present evidence for involvement of Rnr3 in a dNTP independent mitochondrial function(s).

## RESULTS

### Carbon source dependent regulation of Rnr1 and Rnr3

**Figure 1:**
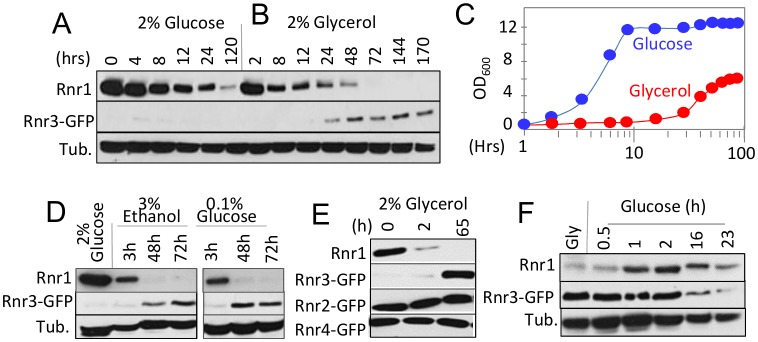
FIGURE 1: Impact of carbon source on the steady state levels of Rnr1 and Rnr3. **(A, B**) *RNR3-GFP* cells were grown to mid-log phase in synthetic complete medium supplemented with 2% glucose (SCD). The cells were collected and released into either SCD (A) or SC medium supplemented with 2% glycerol (SCG; B) for further incubation. Whole cell extract (WCE) samples were prepared at the indicated time points and subjected to Western blot analysis using α-Rnr1 and α-GFP antibodies. Tubulin was used as a loading control.** (C)** OD_600_ of the SCD (blue) and SCG (red) cultures in panels A and B at the indicated time points.** (D)** *RNR3-GFP* cells were grown to mid-log phase in SCD. The cells were collected and released into SC medium supplemented with either 3% ethanol or 0.1% glucose for further incubation. WCE samples were prepared at the indicated time points and subjected to Western blot analysis using α-Rnr1 and α-GFP antibodies. Tubulin was used as a loading control. **(E)** *RNR2-GFP, RNR3-GFP*, and *RNR4-GFP* strains were subjected to the glucose-to-glycerol medium switch as described in panel B. WCE samples were prepared at the indicated time points and subjected to Western blot analysis using α-Rnr1 and α-GFP antibodies. WCE preparation from the *RNR3-GFP* culture was used for α-Rnr1 Western blot.** (F)** *RNR3-GFP* cells were grown to mid log phase in SCG. Cells were collected and released into SCD. WCE samples were prepared at the indicated time points and subjected to Western blot analysis using α-Rnr1 and α-GFP antibodies. Tubulin was used as a loading control.

*RNR1* and *RNR3* are paralogs that arose from the whole genome duplication (WGD) [16]. An outcome of the duplication was increased glycolytic flux, enabling the post WGD yeasts to meet the demands for ATP independently of mitochondrial respiration or oxidative phosphorylation [17]. This suggests that Rnr1 and Rnr3 might have a metabolism dependent function(s), and that their expression might be controlled by metabolic state of the cell. To address this possibility, we assessed the impact of two different carbon sources, glucose and glycerol, on Rnr1 and Rnr3 expression. Glucose is a fermentable carbon source, which at a standard concentration (2%), promotes rapid fermentative proliferation. Glycerol is a non-fermentable carbon source metabolized via oxidative phosphorylation in the mitochondria. As reported [15], only Rnr1 was observed in a standard synthetic complete medium supplemented with 2% glucose (SCD) while Rnr3 remained undetectable (**Figure 1A**). The cells were collected and released into either fresh SCD or synthetic complete medium supplemented with 2% glycerol (SCG) and subjected to further incubation. Cells in SCD continued to divide (**Figure 1C**). In contrast, the cells transferred to SCG ceased to divide for the first 24 hours (**Figure 1C**). During this period, Rnr1 abundance declined steadily until it became undetectable (**Figure 1B**). Cells began dividing again between 24 and 48 hours (**Figure 1C**). Remarkably, the resumption of cell division was accompanied by an increase in Rnr3 levels while Rnr1 remained undetectable (**Figure 1B**).

To determine whether the apparent Rnr1-to-Rnr3 switch was due to glycerol per se or its impact on cellular metabolism, we tested the effects of ethanol, another non-fermentable carbon source, and limiting concentrations (0.1%) of glucose, which activate respiratory metabolism. Results show that both induced Rnr1 down regulation and Rnr3 induction (**Figure 1D**). We infer that the Rnr1-to-Rnr3 switch is a cellular response to respiratory metabolism. We also tested the impact of carbon sources on Rnr2 and Rnr4, the two regulatory subunits of RNR. In contrast to Rnr1 and Rnr3, their expression was unaffected (**Figure 1E**).

The fact that Rnr1 expression is dominant over Rnr3 in glucose medium (e.g. **Figure 1A**) implies that the effects of respiratory carbon source are reversible. To test this, we performed a reciprocal medium switch experiment, from 2% glycerol to 2% glucose. Results show a notable increase in Rnr1 levels by 1 hour following the switch. And by 16 hours, a reduction in Rnr3 is also apparent (**Figure 1F**).

Taken together, these results unveil a carbon source dependent regulation of the two catalytic subunits of budding yeast RNR. Non-fermentable carbon sources or limiting concentrations of glucose, which activate respiratory metabolism, down regulate Rnr1 and induce Rnr3 expression. Oppositely, abundant glucose, which facilitates rapid fermentative proliferation, induces Rnr1 expression and down regulates Rnr3.

### Mec1 mediates both the carbon source dependent induction and down regulation of Rnr3

**Figure 2:**
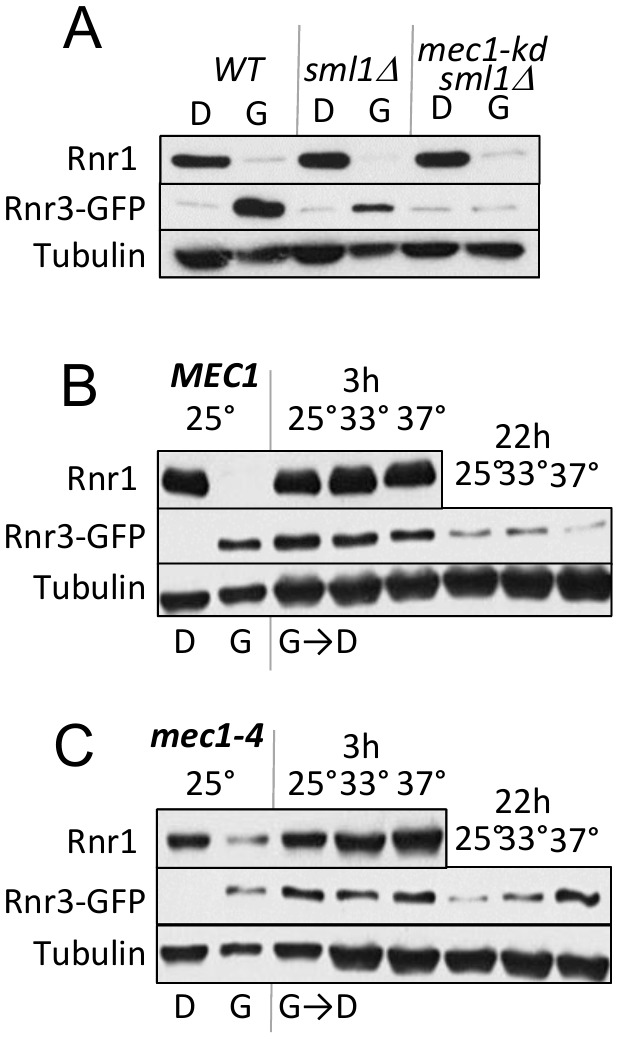
FIGURE 2: Mec1 promotes the carbon source dependent regulation of Rnr3. **(A)** *WT, sml1*Δ, and *mec1-kd sml1*Δ cells were grown to mid-log phase in YPD (“D”) or YPG (“G”). WCE samples were prepared and subjected to Western blot analysis using α-Rnr1 and α-GFP antibodies. Tubulin was used as a loading control. **(B, C)** WT and *mec1-4* cells were grown to mid log phase in YPD (D) or YPG (G) at 25°C, a permissive temperature for *mec1-4* [8]. WCE samples were prepared and subjected to α-Rnr1 and α-GFP Western blot analysis. “G →D”: Cells grown to mid-log phase in YPG were collected and released into YPD and incubated at three different temperatures, 25°C, 30°C, and 37°C. WCE samples were prepared at 3- and 22-hours after the medium switch and subjected to Western blot analysis using α-Rnr1 and α-GFP antibodies. Tubulin was used as a loading control.

As mentioned above, Mec1 mediates the DDR dependent induction of Rnr3 [11, 13]. To test its involvement in the carbon source dependent regulation, we assessed the impact of a kinase dead allele, *mec1-kd*, which remains viable in a *sml1*∆ suppressor mutation background [18]. *WT, mec1-kd sml1*∆, and *sml1*∆ strains were cultured in rich 2% glucose (YPD) or 2% glycerol (YPG) medium until they were in mid-log phase. Protein-extracts were prepared from each culture and analyzed by Western blot using antibodies against Rnr1 and Rnr3-GFP (**Figure 2A**). Glycerol dependent Rnr3 expression was abolished in the *mec1-kd sml1*∆ strain, indicating that it was *MEC1* dependent (**Figure 2A**). In contrast, *MEC1* was dispensable for the carbon source dependent down regulation of Rnr1 (**Figure 2A**).

Next, we wished to address whether Mec1 might also mediate the glucose dependent down regulation of Rnr3 (e.g. **Figure 1F**). However, because the glycerol dependent Rnr3 expression is absent in *mec1-kd sml1*Δ cells (**Figure 2A**), the strain would not be suitable for the experiment. Instead, we utilized a separation of function allele, *mec1-4*, which is viable but confers sensitivity to DNA damage, replication stress, as well as to elevated temperature [8]. This strain, in contrast to the *mec1-kd sml1*∆, is proficient in glycerol dependent Rnr3 induction at 25°C, a permissive temperature (**Figure 2C**).

*WT* and *mec1-4* cells in YPD were released into YPG and subjected to further incubation at 25°C, 33°C and 37°C, a permissive, semi-permissive, and restrictive temperature for *mec1-4*, respectively. Robust increase in Rnr1 was observed by 3 hours in both the *WT* and *mec1-4* strains, irrespective of the temperature (**Figure 2B, C**). By 22 hours, a notable reduction in Rnr3 was observed in the *mec1-4* at 25°C, but not at 37°C, a restrictive temperature for *mec1-4* (**Figure 2C**). The latter shows that the glucose dependent down regulation of Rnr3 is also mediated by Mec1.

### Carbon source dependent Rnr3 induction is independent of the DNA damage response network

**Figure 3:**
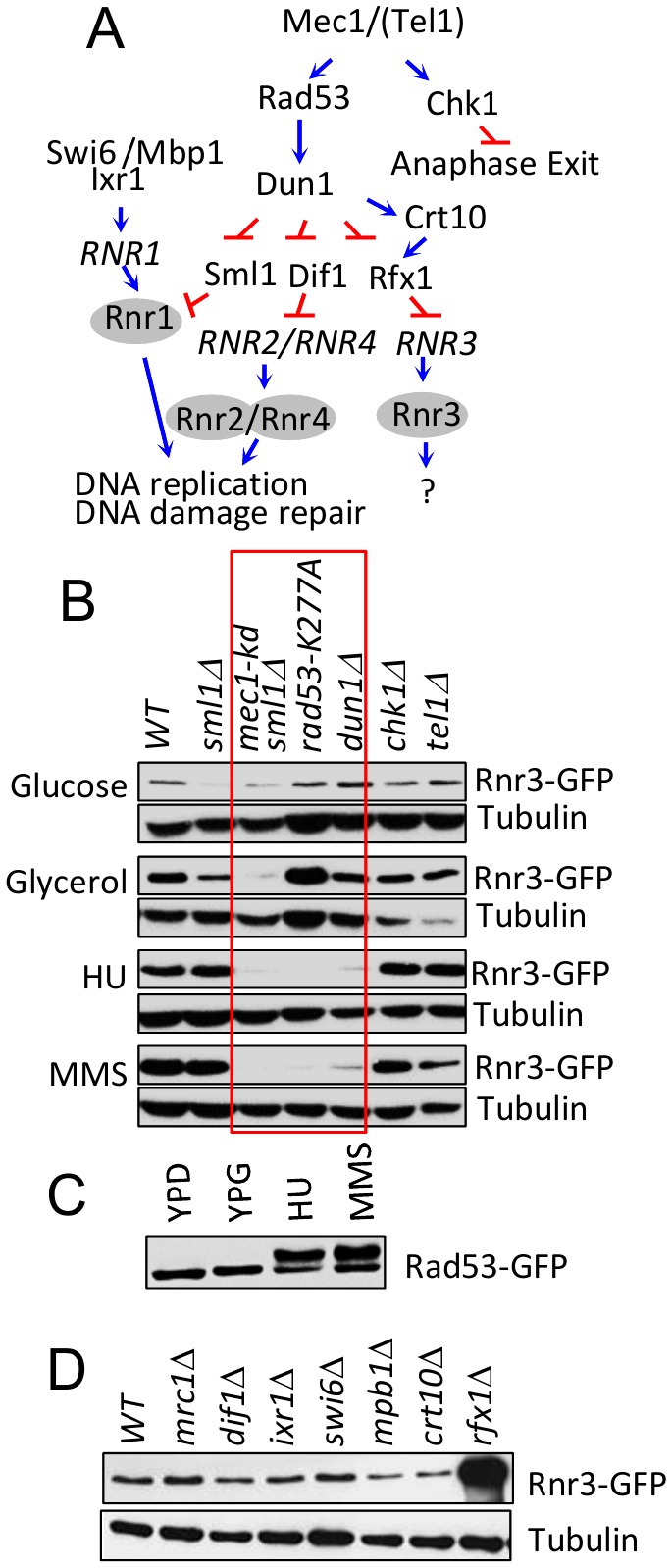
FIGURE 3: Mec1 mediates the carbon source dependent induction of Rnr3 independently of its DNA damage response network. **(A)** Key components of the Mec1 checkpoint response network involved in RNR regulation [19]. Blue arrows and red bars denote a positive and inhibitory impact on the indicated gene or protein. **(B)** Strains of the indicated genotypes (Table S1) were cultured in YPD, YPG, and YPD supplemented with HU (10 mM) or MMS (0.01%). WCE samples were prepared from cells in log phase (YPD and YPG) or following 2 hours exposure to HU or MMS and subjected to Western blot analysis using α-GFP antibodies. Tubulin was used as a loading control. Red box: Mutations that abolish the DDR dependent Rnr3 induction.** (C)** *RAD53-GFP* cells were cultured in YPD, YPG, and YPD supplemented with HU (10 mM) or MMS (0.01%). WCE samples were prepared from cells in log phase (YPD and YPG) or following 2 hours exposure to HU or MMS and subjected to Western blot analysis using α-GFP antibodies.** (D)** WCE samples prepared from the indicated strains (Table S1) grown to mid-log phase in YPG were subjected to Western blot analysis using α-GFP antibodies. Tubulin was used as a loading control.

Rnr3 expression during the DDR is mediated by the Mec1-Rad53-Dun1 signaling axis (**Figure 3A**) [11]. To address involvement of the latter in the carbon source dependent Rnr3 induction, we tested the impact of *rad53-K277A*, a kinase dead allele, and *dun1*∆. Results show that neither mutation had an impact on the glycerol dependent Rnr3 induction, despite the fact that both abolished the HU and MMS dependent induction (**Figure 3B**; red box). This indicates that Mec1 mediates the carbon source dependent Rnr3 induction independently of Rad53 and Dun1. In further support, we find that Rad53 remains unphosphorylated in response to glycerol (**Figure 3C**).

We expanded the analysis to additional components of the Mec1 DDR network: Dif1, Sml1, Swi6, Mbp1, Ixr1, Crt10, Rfx1, Tel1 and Chk1 (**Figure 3A**)[19]. Dif1, Sml1, and Rfx1 are regulators of Rnr2/4, Rnr1, and Rnr3, respectively (above). Tel1 is another budding yeast ATM/ATR protein, which can partially substitute for Mec1 [20]. Chk1 is a serine/threonine kinase that functions to prevents anaphase onset [21]. And Swi6, Mbp1, Ixr1, and Crt10 are involved in regulation of *RNR1* and *RNR3* transcription respectively [19]. Results show that all of the tested mutants are proficient in glycerol dependent Rnr3 induction (**Figure 3B, D**), suggesting that Mec1 mediates the carbon source dependent Rnr3 induction independently of its DDR network.

### The conserved ATP/dATP allosteric regulatory site facilitates efficient removal of Rnr1 in response to carbon source

**Figure 4:**
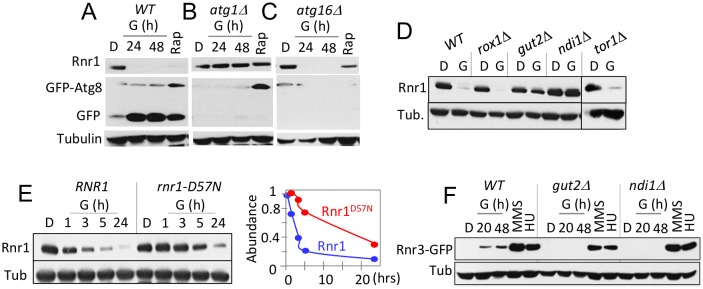
FIGURE 4: Carbon source dependent down regulation of Rnr1 is dependent on oxidative phosphorylation and the conserved ATP/dATP binding site. **(A)** WT cells transformed with a *GFP-ATG8* plasmid was grown to mid log phase in leucine drop out medium supplemented with 2% glucose (SD-LEU; “D”). The cells were collected and transferred to leucine drop out medium supplemented with 2% glycerol (SGLEU; “G”) or SD-LUE2 supplemented with rapamycin (200 ng/ml; “Rap”). WCE samples were prepared from the glycerol culture at 24- and 48-hours after the medium switch or 2 hours in rapamycin, and subjected Western blot analysis using α-Rnr1 and α-GFP antibodies. Tubulin was used as a loading control.** (B, C)** An *atg1*Δ - and *atg16*Δ*-* strains transformed with the *GFP-ATG8* plasmid were subjected to the analysis described in panel (**A**). **(D)** Strains of the indicated genotypes (Table S1) were grown to mid-log phase in YPD (“D”) or YPG (“G”). WCE samples were prepared and subjected to Western blot analysis using α-Rnr1 antibodies. Tubulin was used as a loading control. **(E)** WT and *rnr1-D57N* cells were grown to mid log phase in YPD (“D”). Cells were collected and released into YPG (“G”) for further incubation. Left hand panel: Western blot analysis using α-Rnr1 antibodies. Tubulin was used as a loading control. Right hand side: The Rnr1 signal in each lane was normalized to the tubulin signal in the same lane. The value obtained at each time point was expressed as a fraction of the value at t=0.** (F)** WT, *gut2*Δ, and *ndi1*Δ strains were cultured in YPD (“D”), YPG (“G”), and YPD supplemented with HU (10 mM) or MMS (0.01%). WCE samples in YPD were from cells in mid log phase. The YPG samples were prepared at 20 and 48 hours after medium switch to YPG. The HU and MMS samples were prepared following 2 hour exposure to the respective chemical. WCE samples were subjected to Western blot analysis using α-GFP antibodies. Tubulin was used as a loading control.

Two lines of evidence suggest that the glycerol dependent Rnr1 down regulation might be via autophagy; (i) autophagy regulates Rnr1 abundance in response to genotoxic stress [22] and (ii) budding yeasts activate autophagy in response to non-fermentable carbon source [23]. We wished to test this possibility. First, we utilized GFP-ATG8 as a readout [23], to confirm the glycerol dependent autophagy in our experimental system. Results confirmed a marked increase in GFP, the autophagic cleavage product of the GFP-Atg8 in response to glycerol (**Figure 4A**). Next, we assessed the impact of *atg1*Δ. *ATG1* encodes for the serine/threonine kinase subunit of the Atg1 signalling complex required for autophagy [24]. The mutation abolished glycerol dependent GFP accumulation, confirming that autophagy was impaired (**Figure 4B**). Crucially, Rnr1 persisted in the mutant, implicating autophagy in the down regulation (**Figure 4B**). To further confirm, we tested additional *ATG* genes; *ATG5, 9, 13, 15, 16*, and *18* required for the essential autophagosomal membrane formation, *ATG17* and *31* for starvation induced autophagy, *ATG32* for mitophagy, and *ATG11, 19*, and *24* for in cytoplasm-to-vacuole (CVT) pathway [25]. Surprisingly, results show that the carbon source dependent Rnr1 down regulation proceeds normally in these mutants, indicating that the down regulation is not via autophagy (**Figure 4C**; data not shown).

Results above imply that the carbon source dependent down regulation of Rnr1 is mediated by an autophagy independent function of Atg1. *ATG1* is notable among autophagy genes for its role(s) in facilitating mitochondrial respiration [26], which suggests that the latter might be involved. To address the possibility, we examined the impact of *gut2*Δ and *ndi1*Δ, which impair oxidative phosphorylation: Gut2 is an integral component of the mitochondrial outer membrane involved in glycerol metabolism and NADH oxidation [27]. Ndi1 is a mitochondrial protein that transfers electrons from NADH to ubiquinone in the respiratory chain [28]. Results show that the glycerol dependent Rnr1 down regulation is abolished in these mutants, consistent with the notion that the down regulation is dependent on mitochondrial respiration (**Figure 4D**). We also assessed the impact of *tor1*Δ and *rox1*Δ. Tor1 (Target of Rapamycin) is a serine/threonine kinase involved in nutrient response [29]. Rox1 is a transcription regulator involved in osmotic and hypoxic stress response [30]. Unlike the *gut2*Δ and *ndi1*Δ strains, *tor1*Δ and *rox1*Δ cells are proficient in glycerol dependent Rnr1 down regulation (**Figure 4D**), suggesting that the impact of glycerol is largely due to oxidative phosphorylation irrespective of other effects of the carbon source.

*gut2*Δ and *ndi1*Δ cells are also impaired the glycerol dependent Rnr3 induction; in contrast, both are proficient in HU or MMS dependent Rnr3 induction (**Figure 4F**). The latter provides further support for the proposal that the DDR- and carbon source-dependent Rnr3 induction is mediated by distinct mechanisms.

An essential function of oxidative phosphorylation is ATP production. Notably, ATP is a key regulator of RNR, whose binding to the conserved ATP/dATP allosteric site in the R1 subunit activates the enzyme [1]. This suggests that the effects of respiratory carbon source might be linked to the increased ATP production and its impact on the ATP/dATP binding site. To address this possibility, we examined an allele where the ATP/dATP binding site has been mutated, the *rnr1-D57N* [31]. A notable delay in Rnr1 down regulation was observed in the mutant, consistent with the notion that the down regulation is dependent on ATP levels (**Figure 4E**). The latter also suggests that Rnr1 may function as a sensor for metabolic changes in the cell, facilitating its own down regulation.

### Novel genetic interactors implicate Rnr3's involvement in cellular metabolism

**Figure 5:**
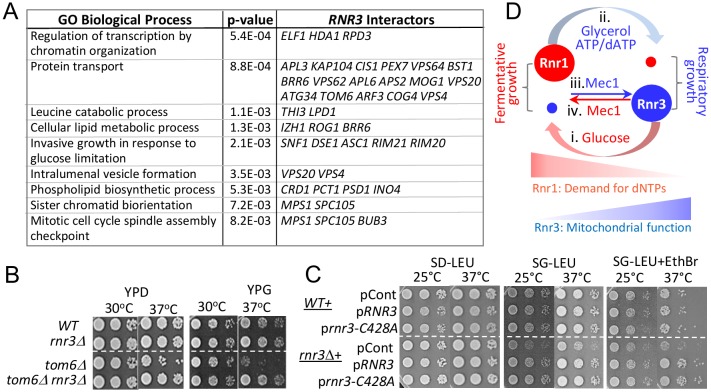
Novel genetic interactors unveil a functional link between Rnr3 and mitochondria. (**A**) The SGA database lists 130 genetic interactors of *RNR3* (Tables S2) [32]. Functional Specification (FunSpec) analysis [39] unveils enrichment of several GO Biological Process terms (Tables S3). **(B)** Ten fold serial dilutions of the indicated strains were spotted onto YPD or YPG and incubated at the indicated temperatures. **(C)** WT and *rnr3*Δ cells transformed with a *RNR3-, rnr3-C428A-*, or an empty LEU2 vector plasmid were spotted onto SD-LEU, SG-LEU, or SG-LEU plus EthBr (0.5 µg/ml) and incubated at the indicated temperatures. **(D)** Model: Mec1 and carbon source dependent regulation of Rnr1 and Rnr3 facilitates fermentative and respiratory growth. (i) Abundant glucose induces Rnr1 in preparation for rapid proliferation and a greater demand for dNTPs. (ii) Glucose depletion or non fermentable carbon sources down regulate Rnr1 as a means to attenuate dNTP production in preparation for slowed growth under respiratory conditions. (iii) Mec1 mediates the respiratory carbon source dependent induction of Rnr3 independently of its DDR network. (iv) Mec1 also facilitates the glucose dependent down regulation of Rnr3 via a mechanism yet to be elucidated.

Synthetic genetic array (SGA) analysis is a high throughput platform for identifying genetic interactors of a gene of interest; moreover, it is a powerful means to uncover a novel function(s) of the gene [32]. We examined the SGA database and found 130 genetic interactors of *RNR3* (Table S2) [32]. Despite the fact that Rnr3 is a catalytic subunit of RNR, whose expression is induced in response to genotoxic stress, there was no enrichment of gene ontology (GO) terms directly linked to DNA synthesis or the DDR (Table S3). Instead, they were enriched for several extra nuclear processes such as “protein transport”, “leucine catabolic process” and “cellular lipid metabolic processes” (**Figure 5A**; Tables S3).

Among the *RNR3* interactors associated with the GO term “protein transport” is *TOM6* (**Figure 5A**; Table S2, S3). Tom6 is a component of the mitochondrial TOM (translocase of outer membrane) complex, responsible for mitochondrial protein import [33]. We find that *tom6*Δ confers temperature sensitivity, which becomes more pronounced under respiratory conditions (**Figure 5B**; compare YPD versus YPG). Remarkably, *rnr3*Δ rescues the *tom6*Δ phenotype, implicating a functional link between Rnr3 and mitochondria (**Figure 5B**). Notably, the rescue was observed under both respiratory (YPG) and fermentative conditions (YPD). This was unexpected given that Rnr3 is undetectable during fermentative proliferation (e.g. **Figure 1A**). We infer that Rnr3 is not only expressed under fermentative conditions, but also facilitates an essential mitochondrial function(s).

In budding yeast, mitochondrial DNA is dispensable under fermentative conditions [35]. Therefore, the *rnr3*Δ rescue of *tom6*Δ growth defects, which manifests under both fermentative and respiratory conditions, suggests that the rescue is independent of mitochondrial genome maintenance and, by extension, dNTP production. To confirm, we assessed the impact of an allele, *rnr3-C428A*, carrying a mutation at a conserved cysteine residue in the active site. The corresponding mutation in Rnr1, the *rnr1-C428A*, is a catalytically dead allele [36]. We introduced the *rnr3-C428A* plasmid marked with LEU2 into a WT and *rnr3*Δ strains and assessed the impact in leucine drop out medium supplemented with either 2% glucose (SD-LEU) or 2% glycerol (SG-LEU) (**Figure 5C**). Surprisingly, we find that *rnr3*Δ cells on leucine drop out plates, unlike the YP plates, exhibit a notable growth defects in glycerol (**Figure 5C**). While the impact of leucine drop-out medium was unexpected, it is consistent with the enrichment of genes associated with the GO term “leucine catabolic process” among the *RNR3* genetic interactors (**Figure 5A**).

The glycerol sensitivity of *rnr3*Δ cells was exacerbated by addition of ethidium bromide (EthBr), a promoter of mitochondrial genome loss, and elevated temperature (**Figure 5C**). Introduction of *RNR3* rescued the phenotype confirming that it was due to the absence of Rnr3 (**Figure 5C**). Importantly, the *rnr3-C428A* plasmid also rescued the phenotype (**Figure 5C**), in support for the proposal that the impact of Rnr3 under respiratory conditions is independent of dNTP.

## DISCUSSION

We present evidence for a carbon source dependent regulation of the budding yeast RNR that impacts expression of the two catalytic subunits, Rnr1 and Rnr3. Non-fermentable carbon sources or limiting concentrations of glucose down regulate Rnr1 and induce Rnr3 expression. Oppositely, abundant glucose induces Rnr1 expression and down regulates Rnr3. While the mechanistic basis and physiological significance are yet to be elucidated, results above provide several insights.

Mec1, an essential ATM/ATR checkpoint response kinase, mediates both the respiratory carbon source dependent induction and glucose dependent removal of Rnr3. Surprisingly, Mec1 mediates the effects independently of the key known components of its DDR network, including Rad53 and Dun1. Our findings implicate a novel Mec1 signalling axis.

We present three lines of evidence for involvement of Rnr3 in cellular metabolism; (i) Analysis of *RNR3* genetic interactors in the SGA database unveils enrichment of genes linked to metabolic processes (**Figure 5A**; Table S3). (ii) *rnr3*Δ confers respiratory growth defects when cultured in leucine drop out medium (**Figure 5C**). And (iii) *rnr3*Δ rescues temperature sensitivity of *tom6*Δ (**Figure 5B**). The latter two suggest a role of Rnr3 in mitochondrial function. In support, two independent studies on mitochondrial proteome analyses found that Rnr3 localizes to the organelle [37, 38].

Deletion of *RNR3* rescues *tom6*Δ phenotype under both respiratory and fermentative conditions, indicating that the rescue is independent of mitochondrial genome maintenance (**Figure 5B**). And a *rnr3* allele carrying a mutation at a critical cysteine residue in the active site, *rnr3-C428A*, recues respiratory growth defects of *rnr3*Δ (**Figure 5C**). Together, these results suggest that Rnr3 has a dNTP independent mitochondrial function. This is consistent with the findings that Rnr3 is the sole component of the yeast RNR found in mitochondria [37, 38].

Three lines of evidence suggest that the respiratory of carbon source dependent down regulation of Rnr1 is linked to oxidative phosphorylation and the ensuing changes in intracellular ATP/dATP ratio: (i) *ATG1*, involved in activation of mitochondrial respiration, is the only *ATG* gene among the tested to be required for Rnr1 down regulation. (ii) Mutations that impair oxidative phosphorylation, *gut2*Δ and *ndi1*Δ, abolish the down regulation. And (iii) down regulation of Rnr1^D57N^, carrying a mutation at the conserved ATP/dATP allosteric regulatory site, is markedly delayed. Intriguingly, the latter suggests that Rnr1 may function as a sensor for changes in metabolic state via its ATP/dATP binding site.

The dramatic reduction in Rnr1 abundance suggests that the gene might be dispensable under respiratory conditions. On the contrary, we find that *rnr1*Δ spores from a *rnr1*Δ/+ diploid strains are unable to form a colony on YPG plate (data not shown). We infer that that Rnr1 is expressed under respiratory conditions at a low level to promote the essential dNTP production.

Taken together, we propose the following model (**Figure 5D**): The carbon source dependent regulation of Rnr1 is a mechanism to control RNR activity to couple dNTP production to differential demands for the DNA building blocks. In the presence of abundant glucose, which facilitates rapid fermentative proliferation, Rnr1 is induced to meet the increased demand for dNTPs. In response to a non-fermentable carbon source or glucose depletion, which activates slower respiratory proliferation, Rnr1 level drops dramatically to prevent accumulation of excessive dNTPs, which can lead to mutator phenotype and/or hinder cell cycle progression [31]. The carbon source dependent regulation of Rnr3, on the other hand, is a mechanism to activate a dNTP independent function of Rnr3, which becomes more critical under respiratory conditions. Mec1, an essential ATM/ATR protein kinase, mediates both the carbon source dependent induction and removal of Rnr3, independently of its DDR network (**Figure 5D**). These findings provide a new framework in understanding the function(s) of Rnr3 and Mec1.

## MATERIALS AND METHODS

### Yeast strains and media

All strains were of either BY or S288C background, except for *mec1-kd RNR3-GFP* and *rad53-K277A RNR3-GFP*, which were generated via crossing a BY and SK1 strain (Table S1). Synthetic complete (SC) media were prepared as described [40] with one of the following carbon sources; 2% (w/v) glucose, 0.1% (w/v) glucose, 2% (v/v) glycerol, or 3% (v/v) ethanol. HU and MMS media were YPD (1% yeast extract, 2% bacto peptone, 2% glucose) supplemented with 10 mM HU or 0.01% (v/v) MMS, respectively.

### Yeast cultures

All liquid cultures were incubated at 30°C unless noted. For a carbon source switching experiment, a 5 ml overnight culture in 2% glucose was diluted to OD_600_ of 0.2 or less on the following morning. The culture was incubated further for ~5 hours until the OD_600_ reached ~0.5. Cells were washed once in H_2_O and resuspended in the medium of choice and incubated further. For glycerol-, ethanol-, or 0.1% glucose cultures, the volume was kept at 20% of the flask size (e.g. 50 ml culture in a 250 ml flask) and shaken at 180 rpm to ensure adequate aeration.

### *rnr3-C428A* plasmid construction

For the construction of the plasmids p*RNR3* and *pRNR3-C428A*, the *RNR3* ORF (±500 bp) was amplified by PCR using genomic DNA from a *rnr1*Δ mutant as a template. The primers used for the amplification of the endogenous *RNR3* include sequences upstream and dowstream of the *RNR3* ORF (bold) and sequences complementary to the vector pRS315, upstream the Pst1 restriction site (Primer A) and downstream the HindIII restriction site (Primer B) (Primer A: 5′-GGCGGCCGCTCTAGAACTAGTGGATCCCCCGGGCTGCAGG **GGCT TGTTTCAGTTTGAACT**-3′ and Primer B: 5′-ACCGGG CCCCCCCTCGAGGTCGACGGTATCGATAAGCTTG **AATTCAATGCT AAATGGTC**-3′). For the construction of p*RNR3-C428A*, primers containing the mutation *C428A* were designed (Primer C: 5′-ATTCGACGATTTCACA***GGC***TAAATTAGATGATTTGAT-3', and Primer D: 5′-ATCAAATCATCTAATTTA***GCC***TGTGAA ATCGTC GAAT-3′). The amplification of the *rnr3* allele carrying the C468A alteration was carried out using the combination of Primers A and C, and Primers B and D. The cloning of the amplified sequences into the plasmid was mediated by gap repair in yeast: For the construction of p*RNR3*, a *rnr3*Δ strain was transformed with the Pst1/HindIII digested pRS315 and the *RNR3* fragment amplified using primers A and B. For the construction of p*rnr3-C428A*, the *rnr3-C428A* fragment amplified using primers C and D were transformed instead. Transformants were selected in leucine drop out synthetic media. Plasmids from the transformants were extracted, amplified in *E. coli*, and the introduction of the mutation was confirmed by DNA sequencing analysis.

### Spot test

Ten-fold serial dilutions from a mid-log culture (10^7 ^cells/ml) were prepared and spotted onto indicated agar plates. Once dry, the agar plates were incubated at the indicated temperature for 1-3 day (2% glucose) or 4-7 (2% glycerol) days before images were taken.

### Western blot analysis

Western blot analysis was performed on TCA (trichloroacetic acid) extracts prepared from culture volumes corresponding to 10 OD_600_ units. Protein extracts were precipitated and pellets were resuspended in loading buffer. After boiling, samples were loaded on 7.5% polyacrylamide gels. GFP tagged proteins (Rnr3, Rnr2, Rnr4, Rad53) were detected with mouse α-GFP antibody (Roche 11814460001, 1:5000). Rnr1 was detected with rabbit α-Rnr1 antibody (Agrisera AS16 3639, 1:10000). Rabbit α-tubulin antibody was used for the detection of tubulin, our loading control (Abcam ab184970, 1:20000). Secondary antibodies used were goat α-Mouse IgG H&L (Abcam Ab6789, 1:10000) and α-Rabbit IgG (Cell Signalling #7074, 1:5000). Secondary antibodies were detected using Western Lightning ECL Pro (PerkinElmer).

## SUPPLEMENTAL MATERIAL

Click here for supplemental data file.

All supplemental data for this article are available online at http://www.microbialcell.com/researcharticles/2019a-corcoles-saez-microbial-cell/.

## Abbreviations

DDR: DNA damage response
GO: gene ontology
RNR: ribonucleotide reductase
SGA: synthetic genetic array
TOM: translocase of outer membrane
WGD: whole genome duplication
