# Supplementary material for: Functional link between mitochondria and Rnr3, the minor catalytic subunit of yeast ribonucleotide reductase
Source: Microb Cell. 2019 May 20;6(6):286–94. doi: 10.15698/mic2019.06.680 (PMC6545439; doi:10.15698/mic2019.06.680)
Supplement: Supplementary file 1 [file mic-06-286-s01.pdf]

Table S1. Strains utilized in current study

Strain	Relevant Genotype	Source
4682	WT (*his3Δ1; leu2Δ0; met15Δ0; ura3Δ0*)	EUROSCARF
4683	*RNR3-GFP::HIS3; his3Δ1; leu2Δ0; met15Δ0; ura3Δ0*	EUROSCARF
835	*RNR2-GFP::KanMX4; can1Δ; ade5Δ; leu2Δ; trp1Δ; ura3Δ*	
836	*RNR4-GFP::KanMX4; can1Δ; ade5Δ; leu2Δ; trp1Δ; ura3Δ*	
4693	*sml1Δ::KanMX4; RNR3-GFP::HIS3; his3Δ1; leu2Δ0; lys2Δ0; ura3Δ0*	This study
4751	*MEC1::mec1-kd1; sml1::hphMX4; RNR3-GFP::HIS3; his3Δ1; met15Δ0; ura3Δ; leu2Δ0;*	This study
4770	*MEC1::mec1-4-URA3 RNR3-GFP::HIS3; his3Δ1; leu2Δ0; met15Δ0*	This study
4514	*rad53K277A-URA3 RNR3-GFP::HIS3 his3Δ1; leu2Δ0; met15Δ0; ura3Δ0*	This study
4713	*dun1Δ::KanMX4; RNR3-GFP::HIS3; his3Δ1; leu2Δ0; lys2Δ0; ura3Δ0*	This study
4694	*chk1Δ::KanMX4; RNR3-GFP::HIS3 his3Δ1; leu2Δ0; lys2Δ0; ura3Δ0 met15Δ0*	This study
4685	*tel1Δ::KanMX4; RNR3-GFP::HIS3; his3Δ1; leu2Δ0; ura3Δ0*	This study
4221	*RAD53-GFP::HIS3; his3Δ1; leu2Δ0; met15Δ0; ura3Δ0*	This study
4696	*mrc1Δ::KanMX4; RNR3-GFP::HIS3; his3Δ1; leu2Δ0; lys2Δ0; ura3Δ0*	This study
4708	*dif1Δ::KanMX4; RNR3-GFP::HIS3; his3Δ1; leu2Δ0; lys2Δ0; ura3Δ0*	This study
4699	*irx1Δ::KanMX4; RNR3-GFP::HIS3; his3Δ1; leu2Δ0; lys2Δ0; ura3Δ0*	This study
4702	*swi6Δ::KanMX4; RNR3-GFP::HIS3; his3Δ1; leu2Δ0; lys2Δ0; ura3Δ0*	This study
4688	*mbp1Δ::KanMX4; RNR3-GFP::HIS3; his3Δ1; leu2Δ0; met15Δ0; ura3Δ0*	This study
4705	*crt10Δ::KanMX4; RNR3-GFP::HIS3; his3Δ1; leu2Δ0; met15Δ0; ura3Δ0*	This study
4710	*rfx1Δ::KanMX4; RNR3-GFP::HIS3; his3Δ1; leu2Δ0; met15Δ0; ura3Δ0*	This study
5345	*atg1Δ::KanMX4 pGFP-ATG8 (LEU2) his3Δ1; leu2Δ0; met15Δ0; ura3Δ0*	This study
5351	*atg16Δ::KanMX4 pGFP-ATG8 (LEU2) his3Δ1; leu2Δ0; met15Δ0; ura3Δ0*	This study
4788	*rox1Δ::KanMX4; his3Δ1; leu2Δ0; lys2Δ0; ura3Δ0*	EUROSCARF
4785	*gut2Δ::KanMX4; his3Δ1; leu2Δ0; lys2Δ0; ura3Δ0*	EUROSCARF
4781	*ndi1Δ::KanMX4; his3Δ1; leu2Δ0; lys2Δ0; ura3Δ0*	EUROSCARF
4787	*tor1Δ::KanMX4; his3Δ1; leu2Δ0; lys2Δ0; ura3Δ0*	EUROSCARF
5443	*rox1Δ::kanMX4; RNR3-GFP::HIS3; his3Δ1; leu2Δ0; met15Δ0; ura3Δ0*	This study
5020	*gut2Δ::KanMX4; RNR3-GFP::HIS3; his3Δ1; leu2Δ0; met15Δ0; ura3Δ0*	This study
5016	*ndi1Δ::KanMX4 RNR3-GFP::HIS3 his3Δ1; leu2Δ0; met15Δ0; ura3Δ0*	This study
5006	*RNR1 CAN1 ade2-1 can1-100 his3-11,15 leu2-3,112 trp1-1 ura3-1 RAD5+*	Chabes A. et al., 2003
5007	*rnr1-D57N CAN1 ade2-1 can1-100 his3-11,15 leu2-3,112 trp1-1 ura3-1 RAD5+*	Chabes A. et al., 2003
6374	*rnr3Δ::hphMX4; can1Δ::STEpr-Sp_his5 lyp1Δ his3Δ1 leu2Δ0 ura3Δ0 met15Δ0*	This study
5338	*pet127Δ::kanMX4; his3Δ1; leu2Δ0; met15Δ0; ura3Δ0*	EUROSCARF
6378	*rnr3Δ::hphMX4; pet127Δ::kanMX4; can1Δ::STEpr-Sp_his5; lyp1Δ; his3Δ1; leu2Δ0; ura3Δ0; met15Δ0*	This study
5337	*tom6Δ::kanMX4; his3Δ1; leu2Δ0; met15Δ0; ura3Δ0*	EUROSCARF
6380	*rnr3Δ::hphMX4; tom6Δ::kanMX4; can1Δ::STEpr-Sp_his5; lyp1Δ; his3Δ1; leu2Δ0; ura3Δ0; met15Δ0*	This study
4605	*rnr1Δ::kanMX4; his3Δ1; leu2Δ0; met15Δ0; ura3Δ0*	EUROSCARF
6566	WT (4682) pRS315	This study
6567	WT (4682) p*RNR3*	This study
6568	WT (4682) p*RNR3-C428A*	This study
6569	*rnr3Δ::hphMX4* (6374) pRS315	This study
6570	*rnr3Δ::hphMX4* (6374) p*RNR3*	This study
6571	*rnr3Δ::hphMX4* (6374) p*RNR3-C428A*	This study

Table S2. Genetic interactors of *RNR3* [32].

apl3	mrpl39	tus1
apl6	mrpl9	ubp3
aps2	mrx14	ubr1
arf3	mtg1	vam7
arp2	mum2	vps20
asc1	nut1	vps4
atg34	oca5	vps62
atg38	ost4	vps64
brp1	paa1	YAR029W
brr6	pct1	YBL036C
bst1	pet127	YCR102C
bub3	pet18	YDL086W
bud21	pex7	YDL157C
ccw14	pho86	YDR415C
chk1	pol30	YEL020C
cis1	prm7	YGR039W
cog4	psd1	YHR050W-A
coq2	rad14	YMR209C
coq4	rad55	ypc1
crd1	rax1	YPR089W
crs5	rev1	
cyk3	rgc1	
dal80	rgi1	
dbp3	rib2	
dig1	rim20	
dld1	rim21	
dml1	rim9	
dse1	rnp1	
ecm15	rnr4	
eds1	rny1	
elf1	rog1	
env10	rpd3	
erv2	rpl21b	
eug1	rpl43a	
fks1	rpl6b	
frm2	rpl9b	
gcd7	rps6b	
gcv2	rsm22	
gde1	rtt103	
glt1	sam1	
grx4	scl1	
hda1	sgf73	
hyp2	smf3	
icp55	snf1	
ino4	spc105	
itc1	spe3	
izh1	srb6	
kap104	ssp1	
kip3	ste50	
lpd1	swd1	
lre1	sym1	
mdm36	thi3	
mob2	thi4	
mog1	tom6	
mps1	trp4	

Table S3. FunSpec (Functional Specificaion) analysis [39] of *RNR3* genetic interactors

GO Molecular Function

Category	p-value	In Category from Cluster	k	f
glycine dehydrogenase (decarboxylating) activity [GO:0004375]	2.1E-03	LPD1 GCV2	2	4
histone deacetylase activity (H3-K9 specific) [GO:0032129]	3.5E-03	HDA1 RPD3	2	5
RNA polymerase II core binding [GO:0000993]	3.5E-03	RTT103 ELF1	2	5
histone deacetylase activity (H3-K14 specific) [GO:0031078]	3.5E-03	HDA1 RPD3	2	5
histone deacetylase activity (H3-K16 specific) [GO:0034739]	3.5E-03	HDA1 RPD3	2	5
carboxy-lyase activity [GO:0016831]	4.6E-03	THI3 YEL020C PSD1	3	18
NAD-dependent histone deacetylase activity (H4-K16 specific) [GO:0046970]	5.2E-03	HDA1 RPD3	2	6
NAD-dependent histone deacetylase activity (H3-K9 specific) [GO:0046969]	5.2E-03	HDA1 RPD3	2	6
NAD-dependent histone deacetylase activity (H3-K14 specific) [GO:0032041]	5.2E-03	HDA1 RPD3	2	6
thiamine pyrophosphate binding [GO:0030976]	9.5E-03	THI3 YEL020C	2	8

GO Biological Process

Category	p-value	In Category from Cluster	k	f
regulation of transcription by chromatin organization [GO:0034401]	5.4E-04	ELF1 HDA1 RPD3	3	9
protein transport [GO:0015031]	8.8E-04	APL3 KAP104 CIS1 PEX7 VPS64 BST1 BRR6 VPS62 APL6 APS2 MOG1 VPS20 ATG34 TOM6 ARF3 COG4 VPS4	17	379
leucine catabolic process [GO:0006552]	1.1E-03	THI3 LPD1	2	3
cellular lipid metabolic process [GO:0044255]	1.3E-03	IZH1 ROG1 BRR6	3	12
invasive growth in response to glucose limitation [GO:0001403]	2.1E-03	SNF1 DSE1 ASC1 RIM21 RIM20	5	48
intralumenal vesicle formation [GO:0070676]	3.5E-03	VPS20 VPS4	2	5
phospholipid biosynthetic process [GO:0008654]	5.3E-03	CRD1 PCT1 PSD1 INO4	4	37
sister chromatid biorientation [GO:0031134]	7.2E-03	MPS1 SPC105	2	7
mitotic cell cycle spindle assembly checkpoint [GO:0007094]	8.2E-03	MPS1 SPC105 BUB3	3	22

GO Cellular Component

Category	p-value	In Category from Cluster	k	f
AP-2 adaptor complex [GO:0030122]	2.1E-03	APL3 APS2	2	4
glycine cleavage complex [GO:0005960]	2.1E-03	LPD1 GCV2	2	4
membrane coat [GO:0030117]	3.2E-03	APL3 APL6 APS2	3	16

MIPS Functional Classification

Category	p-value	In Category from Cluster	k	f
secondary metabolism [01.20]	1.3E-03	YDL086W COQ4 COQ2	3	12
phospholipid metabolism [01.06.02.01]	1.9E-03	CRD1 BST1 PCT1 PSD1 INO4 GDE1	6	68
regulation of the metabolism of vitamins, cofactors, and prosthetic groups [01.07.07]	3.5E-03	THI3 PAA1	2	5
biosynthesis of vitamins, cofactors, and prosthetic groups [01.07.01]	5.1E-03	THI3 COQ4 LPD1 THI4 COQ2 RIB2 SPE3	7	110
degradation of glycine [01.01.09.01.02]	5.2E-03	LPD1 GCV2	2	6

MIPS Protein Complexes

Category	p-value	In Category from Cluster	k	f

Complex Number 78, probably membrane biogenesis and traffic [550.1.78]	7.6E-04	APL3 APL6 APS2	3	10
AP-2 complex [260.20.20]	2.1E-03	APL3 APS2	2	4
Glycine decarboxylase [200]	2.1E-03	LPD1 GCV2	2	4
Complex Number 292 [550.2.292]	3.5E-03	ARP2 ICP55	2	5
Complex Number 123, probably protein/RNA transport [550.1.123]	6.2E-03	KAP104 SAM1 FKS1	3	20
Complex Number 39, probably intermediate and energy metabolism [550.1.39]	9.5E-03	LPD1 SAM1	2	8

MIPS Protein Classes

Category	p-value	In Category from Cluster	k	f
Ubiquitin-protein ligases (E3) [191.41]	9.5E-03	UBP3 UBR1	2	8

PFam-A Domains

Category	p-value	In Category from Cluster	k	f
<A href='http://pfam.sanger.ac.uk/family?id=Hist_deacetyl'>Hist_deacetyl</A>	3.5E-03	HDA1 RPD3	2	5
<A href='http://pfam.sanger.ac.uk/family?id=TPP_enzyme_M'>TPP_enzyme_M</A>	7.2E-03	THI3 YEL020C	2	7
<A href='http://pfam.sanger.ac.uk/family?id=TPP_enzyme_C'>TPP_enzyme_C</A>	7.2E-03	THI3 YEL020C	2	7
<A href='http://pfam.sanger.ac.uk/family?id=TPP_enzyme_N'>TPP_enzyme_N</A>	7.2E-03	THI3 YEL020C	2	7
<A href='http://pfam.sanger.ac.uk/family?id=Adaptin_N'>Adaptin_N</A>	9.5E-03	APL3 APL6	2	8

MDS Proteomics Complexes

Category	p-value	In Category from Cluster	k	f
YAR007C (RFA1)	3.5E-03	ARP2 ICP55	2	5

Cellzome Complexes

Category	p-value	In Category from Cluster	k	f
YBL037W (APL3)	2.1E-03	APL3 APS2	2	4
YDR062W (LCB2)	2.7E-03	KAP104 SAM1 FKS1	3	15
YDR148C (KGD2)	7.2E-03	LPD1 SAM1	2	7
